# Global Outcome Trajectories up to 10 Years After Moderate to Severe Traumatic Brain Injury

**DOI:** 10.3389/fneur.2019.00219

**Published:** 2019-03-14

**Authors:** Marit V. Forslund, Paul B. Perrin, Cecilie Røe, Solrun Sigurdardottir, Torgeir Hellstrøm, Svein A. Berntsen, Juan Lu, Juan Carlos Arango-Lasprilla, Nada Andelic

**Affiliations:** ^1^Department of Physical Medicine and Rehabilitation, Oslo University Hospital, Oslo, Norway; ^2^Department of Psychology, Virginia Commonwealth University, Richmond, VA, United States; ^3^Institute of Clinical Medicine, Faculty of Medicine, University of Oslo, Oslo, Norway; ^4^Department of Research, Sunnaas Rehabilitation Hospital, Nesoddtangen, Norway; ^5^Department of Physical Medicine and Rehabilitation, Sørlandet Hospital, Kristiansand, Norway; ^6^Department of Family Medicine and Population Health, Division of Epidemiology, Virginia Commonwealth University, Richmond, VA, United States; ^7^Faculty of Medicine, Institute of Health and Society, Research Centre for Habilitation and Rehabilitation Models and Services (CHARM), University of Oslo, Oslo, Norway; ^8^Ikerbasque, Basque Foundation for Science, Bilbao, Spain; ^9^BioCruces Health Research Institute, Cruces University Hospital Barakaldo, Barakaldo, Spain

**Keywords:** brain injury, outcome assessment, GOSE, prospective studies, rehabilitation

## Abstract

**Aims:** Based on important predictors, global functional outcome after traumatic brain injury (TBI) may vary significantly over time. This study sought to: (1) describe changes in the Glasgow Outcome Scale–Extended (GOSE) score in survivors of moderate to severe TBI, (2) examine longitudinal GOSE trajectories up to 10 years after injury, and (3) investigate predictors of these trajectories based on socio-demographic and injury characteristics.

**Methods:** Socio-demographic and injury characteristics of 97 TBI survivors aged 16–55 years were recorded at baseline. GOSE was used as a measure of TBI-related global outcome and assessed at 1-, 2-, 5-, and 10-year follow-ups. Hierarchical linear models were used to examine global outcomes over time and whether those outcomes could be predicted by: time, time^*^time, sex, age, partner relationship status, education, employment pre-injury, occupation, cause of injury, acute Glasgow Coma Scale score, length of post-traumatic amnesia (PTA), CT findings, and Injury Severity Score (ISS), as well as the interactions between each of the significant predictors and time^*^time.

**Results:** Between 5- and 10-year follow-ups, 37% had deteriorated, 7% had improved, and 56% showed no change in global outcome. Better GOSE trajectories were predicted by male gender (*p* = 0.013), younger age (*p* = 0.012), employment at admission (*p* = 0.012), white collar occupation (*p* = 0.014), and shorter PTA length (*p* = 0.001). The time^*^time^*^occupation type interaction effect (*p* = 0.001) identified different trajectory slopes between survivors in white and blue collar occupations. The time^*^time^*^PTA interaction effect (*p* = 0.023) identified a more marked increase and subsequent decrease in functional level among survivors with longer PTA duration.

**Conclusion:** A larger proportion of survivors experienced deterioration in GOSE scores over time, supporting the concept of TBI as a chronic health condition. Younger age, pre-injury employment, and shorter PTA duration are important prognostic factors for better long-term global outcomes, supporting the existing literature, whereas male gender and white collar occupation are vaguer as prognostic factors. This information suggests that more intensive and tailored rehabilitation programs may be required to counteract a negative global outcome development in survivors with predicted worse outcome and to meet their long-term changing needs.

## Introduction

Traumatic brain injury (TBI) remains one of the main causes of life years lost due to disability or death (1, 2). Worldwide, an estimated 50 million people sustain TBI each year (1). Research over past decades has made it clear that TBI should be conceptualized as a chronic health condition as opposed to an acute time bound event, as it continues to evolve long after initial recovery (3, 4).

The level of disability and global neurological functional outcome following TBI is commonly measured with the Glasgow Outcome Scale (GOS) or its extended version (GOSE) (5, 6). Both summarize the overall impact of TBI on function, independence and participation. Currently, the GOSE is the recommended core global measurement in TBI research (7, 8). Several large-scale studies have found that about 50% of individuals achieve a favorable outcome (i.e., moderate disability or good recovery outcome) at 6 months after sustaining moderate to severe TBI (9, 10), while a favorable outcome was assessed in 42% of individuals 6 months after sustaining severe TBI (11). Ponsford et al. (12) assessed GOSE scores using a cross-sectional design approximately 10 years after complicated mild to severe TBI, and found that 52% of individuals had good recovery, 44% had moderate disability and 5% had severe disability.

Thus far, only a few studies have assessed GOSE trajectories over longer periods after TBI (e.g., over 5 years). Corrigan and Hammond (4) examined changes in GOSE score categories over four consecutive follow-ups up to 15 years after TBI with data from the Traumatic Brain Injury Model Systems (TBIMS) database (13) in the US, and found dynamic positive and negative changes in GOSE scores between the time points. A UK study (14) assessed changes in disability from 1 year to 5–7 years after mild to severe TBI, and found that 24% of survivors with moderate to severe disability had improved to good recovery, whereas 25% of survivors with good recovery deteriorated to disabled. A Norwegian study (15) followed up survivors of moderate to severe TBI longitudinally and found that GOSE scores remained stable across the first 5 years after injury. A Swedish study (16) reported no significant difference in GOS outcome between 1 year and 10–15 years in survivors after severe TBI; similarly, a recent Norwegian study (17) found stable global functioning between 10 and 20 years after moderate to severe TBI. These Scandinavian findings of stable levels of disability are contrary to the findings in two large TBIMS studies (18, 19) that reported initial improvement in functional status up to approximately 10 years after injury, followed by a peak and a decline in GOSE scores (i.e., increasing disability).

There is increasing evidence for the factors that predict functional outcome after TBI; age (10, 15, 16, 18–26), sex (20, 27), education (21, 28–30), pre-injury employment (15, 20, 28, 31), race (18, 32, 33), history of alcohol abuse prior to injury (20, 34), presence of intracranial lesions (25, 35–37), acute Glasgow Coma Scale (GCS) score (25, 37), duration of post-traumatic amnesia (PTA) (15, 29), duration of hospitalization and rehabilitation stays (18, 19), executive function and memory problems (14, 21, 29, 30, 38), and mood disorders (14, 21, 29, 34, 39, 40). However, the findings of the predictive power of these factors are mixed, partly due to methodological differences between the studies.

Our research group has published GOSE score trajectories up to 5 years after moderate to severe TBI (15). The present study is an extension with a 10-year follow-up after injury. TBI survivors may live for decades after their injury and a better understanding of long-term global outcome after moderate to severe TBI is needed. Delineating the relationships between socio-demographics and injury severity characteristics and functional outcome may yield valuable information on management, rehabilitation, and counseling for TBI survivors at risk for impaired recovery.

The specific study aims were:

1. To describe GOSE score changes up to 10 years after injury.
2. To assess the trajectories of global functioning in people with moderate to severe TBI at 1, 2, 5, and 10 years post-injury.
3. To investigate whether socio-demographics and injury severity characteristics can predict the trajectories of global functioning.

Based on results from our previous follow-up studies in the first 5 years (15) and 10–20 years after injury (17), we hypothesized that TBI-related global outcome would remain stable over the first 10 years after moderate to severe TBI, and that age, sex, pre-injury employment and injury severity characteristics such as PTA would be associated with functional outcome.

## Materials and Methods

### Participants

The present study is a longitudinal cohort consisting of individuals with TBI who were admitted to the Trauma Referral Centre for the Southeast region of Norway from 2005 to 2007. The participants were assessed in the acute phase (baseline) and followed up at 1, 2, 5, and 10 years after injury. **The inclusion criteria were:** (a) age 16–55 years, (b) admission with ICD-10 diagnosis S06.0-S06.9 within 24 h of injury, (c) moderate to severe TBI, classified by an acute GCS score of 3–12 (41) at admission or before intubation, and (d) residence in eastern Norway. **The exclusion criteria were:** (a) previous neurological disorders/injuries, (b) associated spinal cord injuries, (c) previously diagnosed severe psychiatric or substance abuse disorders, and (d) unknown address or incarceration.

In total, 133 people with TBI fulfilled the inclusion criteria. Of these, 24 died in the acute or post-acute phase, and four withdrew before the 1-year follow-up. One participant died and four dropped out of the study between 1 and 2 years. Between the 2- and 5-year follow-up, two participants died and four dropped out. Between the 5- and 10-year follow-up, 5 participants died and 12 dropped out, leaving 77 participants at the last follow-up. Altogether, 32 individuals died from baseline to 10-year follow-up, and these were excluded from the statistical analyses. The present study analyzed data from the surviving population with complete GOSE data at the 1-year follow-up (*n* = 97), with an attrition rate of 21% from the 1–10-year follow-up. A series of papers on functional outcome and health-related quality of life have previously been published based upon the same longitudinal cohort (15, 31, 42–51), please see Howe et al. (43) for a detailed flowchart of the follow-up process up to 10 years after injury.

### Measures

In the present study, the dependent variable was the GOSE (6). The GOSE measures global outcomes (independence, employment, social and leisure activities, family and friendship, return to normal life) after TBI and divides individuals into the following outcome categories: 1 = dead, 2 = vegetative state, 3 = lower severe disability (i.e., complete dependence on others), 4 = upper severe disability (i.e., dependence on others, but can be on their own for 8 hours), 5 = lower moderate disability (living independently, not working or working at a lower level of performance/sheltered work), 6 = upper moderate disability (returning to previous work with adjustments), 7 = lower good recovery (almost back at full functional recovery; only minor physical or mental deficits), and 8 = upper good recovery (full functional recovery). The following independent variables (predictors) were used in the present study: Sex (male vs. female), age at time of injury (continuous, in years), relationship status at time of injury [partnered (married/cohabitant) vs. single], education at time of injury (continuous in years or categorical, i.e., ≤12 years vs. >12 years), employment status at time of injury (employed vs. unemployed), occupation type at time of injury [blue collar (physical work) vs. white collar (non-physical work/student)], acute GCS score (continuous, range 3–12), cause of injury (traffic accident vs. other), length of PTA (continuous, in number of days) as measured by the Galveston Orientation and Amnesia Test (GOAT) (52), computed tomography (CT) head Marshall scores [grading injury severity from I (no visible intracranial pathology) to VI (non-evacuated mass lesions)] (53) on the “worst” CT scan within the first 24 h of injury (i.e., the scan showing most extensive intracranial damage), and Injury Severity Score (ISS, continuous, ranges 1–75 [best to worst]) (54).

### Procedure

Pre-injury and injury-related variables were extracted from medical records. At the 1-, 2-, 5- and 10-year follow-ups, the assessments of the participants including GOSE were most commonly performed by a physiatrist at the outpatient department. In some cases the assessments were completed by an ambulatory team originating from the outpatient department, or by phone interview, if requested by the participants. All participants provided written informed consent to take part in the study.

### Statistical Analysis

Descriptive statistics were used to present socio-demographics and injury-related variables, and the results are presented as percentages and means with standard deviations (SD) or medians with interquartile range (IQR) as appropriate. GOSE score changes over time were also examined with descriptive statistics.

Hierarchical linear models (HLMs) were used to assess the trajectory of global function and examine baseline predictors of GOSE trajectory architecture across 1, 2, 5, and 10 years after injury. Full information maximum likelihood (FIML) estimation was used for handling missing data at the follow-ups, thus retaining all participants in the model (*n* = 97). A conditional (null) model was run first to determine whether there was sufficiently large clustering of GOSE score variance within participants to proceed with HLM. Unconditional growth (linear), quadratic, and cubic models were then run without predictors to determine the most accurate model for linear or polynomial architecture of GOSE scores over time.

Once the most accurate curvature model was identified, predictors were entered simultaneously as fixed effects into a HLM after being centered or given a reference point of 0, along with time and time^*^time (due to the selection of a quadratic trend of GOSE scores over time, outlined below). The first full model used a HLM to determine whether quadratic trajectories of GOSE scores across the four time points could be predicted by the socio-demographic and injury characteristics of time [coded as 0 (1 year), 1 (2 years), 4 (5 years), or 9 (10 years) to reflect actual spacing between time points], time^*^time, sex (1 = woman, 0 = man), age, partner relationship status (1 = partnered, 0 = single), education, employment at time of injury (1 = employed, 0 = unemployed), occupation type (1 = white collar, 0 = blue collar), GCS score, cause of injury (1 = motor vehicle, 0 = not motor vehicle), length of PTA (days), CT severity score, and ISS. A final HLM included the previously significant predictors from the first full model, time, time^*^time, and the interaction terms between time^*^time and the previously significant predictors.

## Results

The mean age of the 97 participants at the time of injury was 30.3 years (SD = 10.8); 78% of the participants were male. The mean GCS score at hospital admission was 7.2 (SD = 3.2); the mean PTA was 26 days (SD = 30). The mean ISS score was 30.0 (SD = 13.6). Two-thirds of the participants had severe TBI according to GCS score, whereas about half of the participants were classified as having more severe intracranial injury according to the CT head Marshall Score. At time of injury, 83% of the participants were employed and 53% had white collar occupations. Table 1 presents the socio-demographic and injury-related characteristics.

**Table 1:** Socio-demographics at time of injury and injury characteristics of 97 survivors.

**Variable**	***n* (%)**	**Total *n***
Age at injury		97
Mean (SD)	30.3 (10.8)	
Sex		97
Male	76 (78.4)	
Female	21 (21.6)	
Relationship status		97
Partnered	28 (28.9)	
Single	69 (71.1)	
Education level		96
≤12 years	54 (56.3)	
>12 years	42 (43.7)	
Employment status		97
Employed	80 (82.5)	
Unemployed	17 (17.5)	
Occupation type		97
Blue collar	46 (47.4)	
White collar	51 (52.6)	
Injury cause		97
Traffic accident	58 (59.8)	
Other	39 (40.2)	
Glasgow coma scale score		97
Mean (SD)	7.2 (3.2)	
Moderate (9–12)	32 (33.0)	
Severe (3–8)	65 (67.0)	
Post-traumatic amnesia duration		91
Days, Mean (SD)	26.0 (30.0)	
Median (IQR)	18.0 (2–38)	
CT Head Marshall Score		97
Mean (SD)	2.6 (1.1)	
Score 1-2	46 (47.4)	
Score 3+	51 (52.6)	
Injury Severity Score		97
Mean (SD)	30.0 (13.6)	
Total acute length of stay		97
Days, mean (SD)	29.0 (25.0)	
In-patient rehab. length of stay		71^*^
Days, Mean (SD)	59.0 (37.0)	

* *In-patient rehabilitation was received by 71 individuals in total (mean length of stay is only calculated for those receiving it)*.

### GOSE Score Changes Over Time

**Figure 1:**
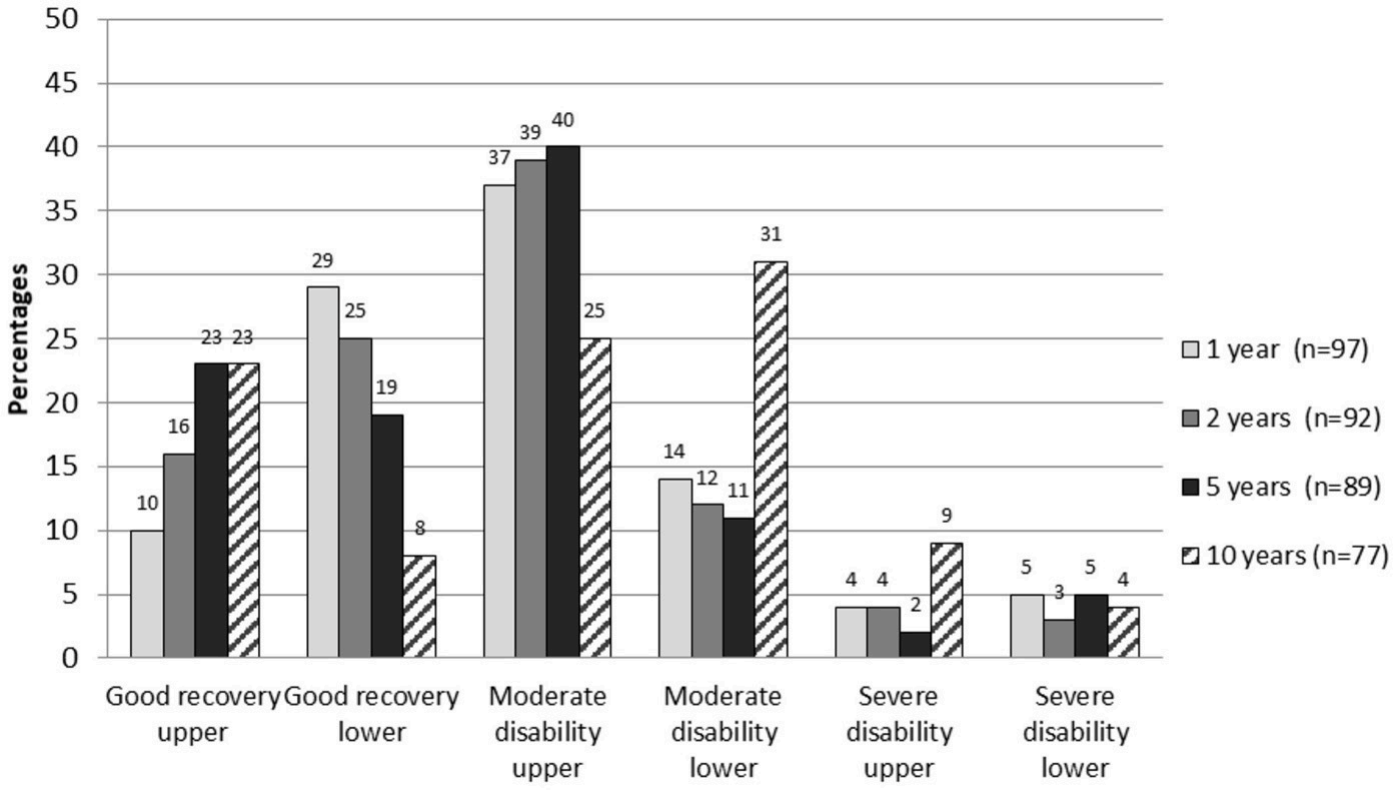
GOSE score distribution at 1-, 2-, 5-, and 10-year follow-up.

Figure 1 shows the distribution of patient frequency between GOSE score categories. The proportion of participants with upper good recovery increased over time from 10 to 23% from 1 year to 10 years after injury, whereas the proportion of participants in the lower good recovery group decreased markedly from 29% to 8%. The trend between the moderate disability categories was the opposite, with the proportion of participants in the upper moderate disability group remaining stable at 37–40% from 1 year to 5 years before decreasing to 25% at the 10-year follow-up, whereas the proportion of participants in the lower moderate disability group approximately doubled from 14 to 31%. The severe disability groups remained relatively stable before there was an increase in the upper severe disability group at the 10-year follow-up.

Table 2 shows the changes in GOSE score categories between the 1- and 2-year, 2- and 5-year, and 5- and 10-year follow-up. The majority of participants had stable GOSE scores between each time point, with 57–67% showing no change. In the 1- to 2-year and 2- to 5-year follow-ups, 21–22% of participants had an increase of one GOSE category, whereas this dropped to 7% in the 5- to 10-year follow-up. Conversely, only 9–13% of participants had a decrease in one category between the 1- to 2-year and 2- to 5-year follow-up, whereas 30% had a decrease between the 5- to 10-year follow-up.

**Table 2:** Changes in Glasgow Outcome Scale-Extended (GOSE) categories (in percentages) between time-points.

**GOSE change**	**1–2 years** **(*n* = 92)**	**2–5 years** **(*n* = 86)**	**5–10 years** **(*n* = 76)**
Increased 2 categories	2	2	0
Increased 1 category	22	21	7
No change	67	62	56
Decreased 1 category	9	13	30
Decreased 2 categories	0	2	7

In total, of the 77 participants with GOSE data at both 1- and 10-year follow-up, 77% had changed GOSE scores between follow-ups (across all time points). Of those with the same GOSE score at 1 and 10 years (*n* = 28), more than one-third had a dynamic GOSE score change between the time points. When only looking at GOSE score changes between the 1- and 10-year follow-up, 26% of participants had increased one, two, or three GOSE categories in terms of function, 36% showed no change, whereas 38% decreased one to two categories (data not shown).

### Unconditional Model and Unconditional Growth Model

The unconditional model yielded a statistically significant estimated participant variance of 1.10 (Wald *Z* = 5.71, *p* < 0.001), and a statistically significant estimated residual variance of 0.83 (Wald *Z* = 11.43, *p* < 0.001). The intraclass correlation coefficient was 0.57, indicating that approximately 57% of the total variance of GOSE scores was associated with participant grouping and that the assumption of independence was violated. This suggests there was sufficiently large clustering of GOSE score variance within participants to proceed with a HLM. The unconditional model was then run separately with the successive additions time, quadratic time, and cubic time to determine the shape of the best-fitting curve of the GOSE over time (Table 3), suggesting that a quadratic trajectory best fit the GOSE over time.

**Table 3:** Model fit for GOSE trajectories over time.

**Model**	**-2 Log Likelihood**
Unconditional growth model	1049.64
Quadratic	1022.20^*^
Cubic	1021.96

Criticalχ^2^ value for significant difference at α = 0.05 is ≥ 3.841 drop from the previous model (

* *= significant improvement)*.

### Full HLM

**Figure 2:**
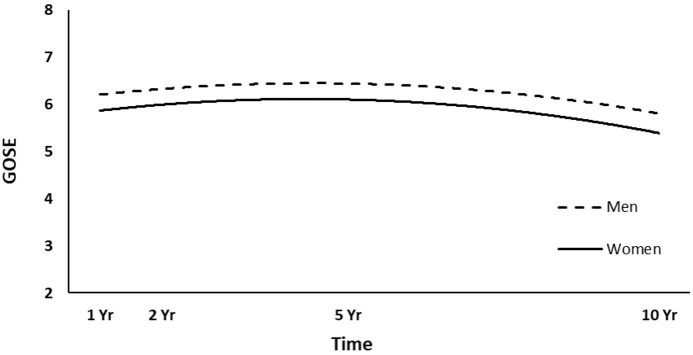
Main effect of sex on GOSE trajectories.

**Figure 3:**
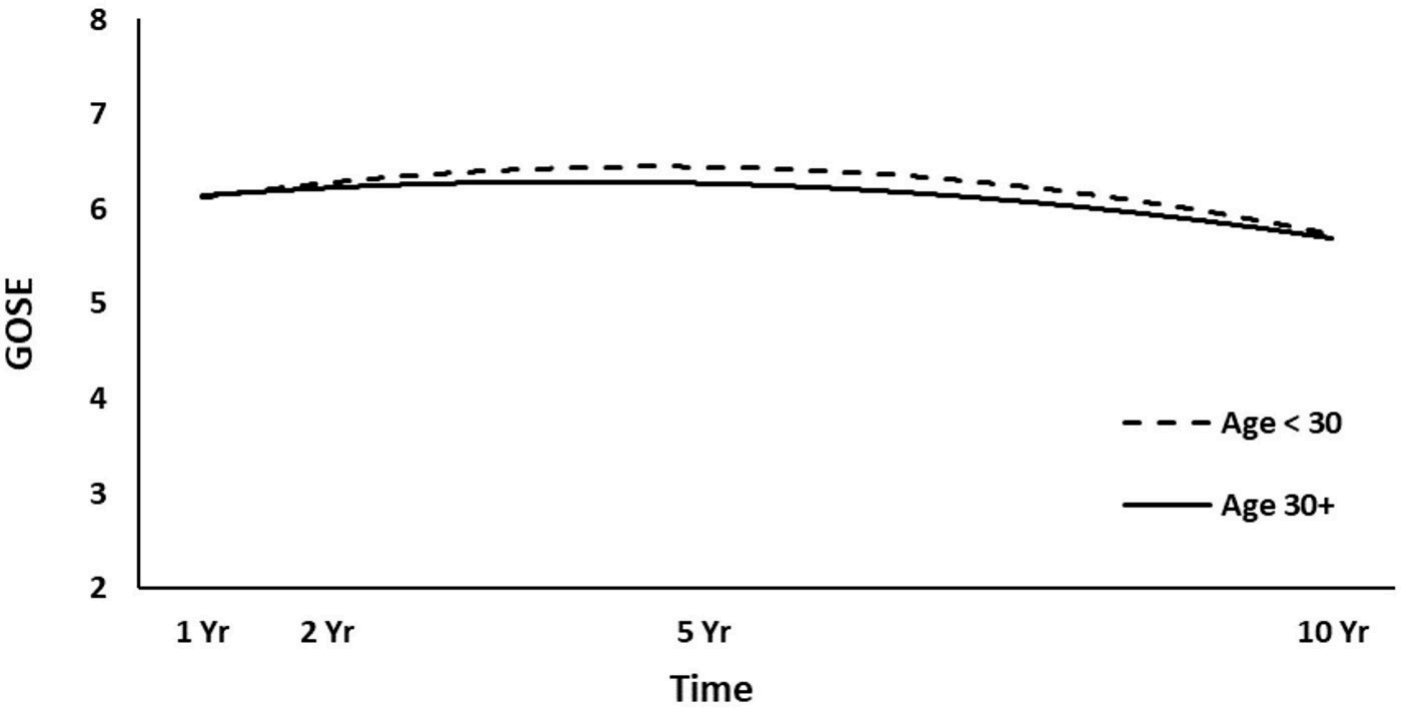
Main effect of age (dichotomized at mean value) on GOSE trajectories.

**Figure 4:**
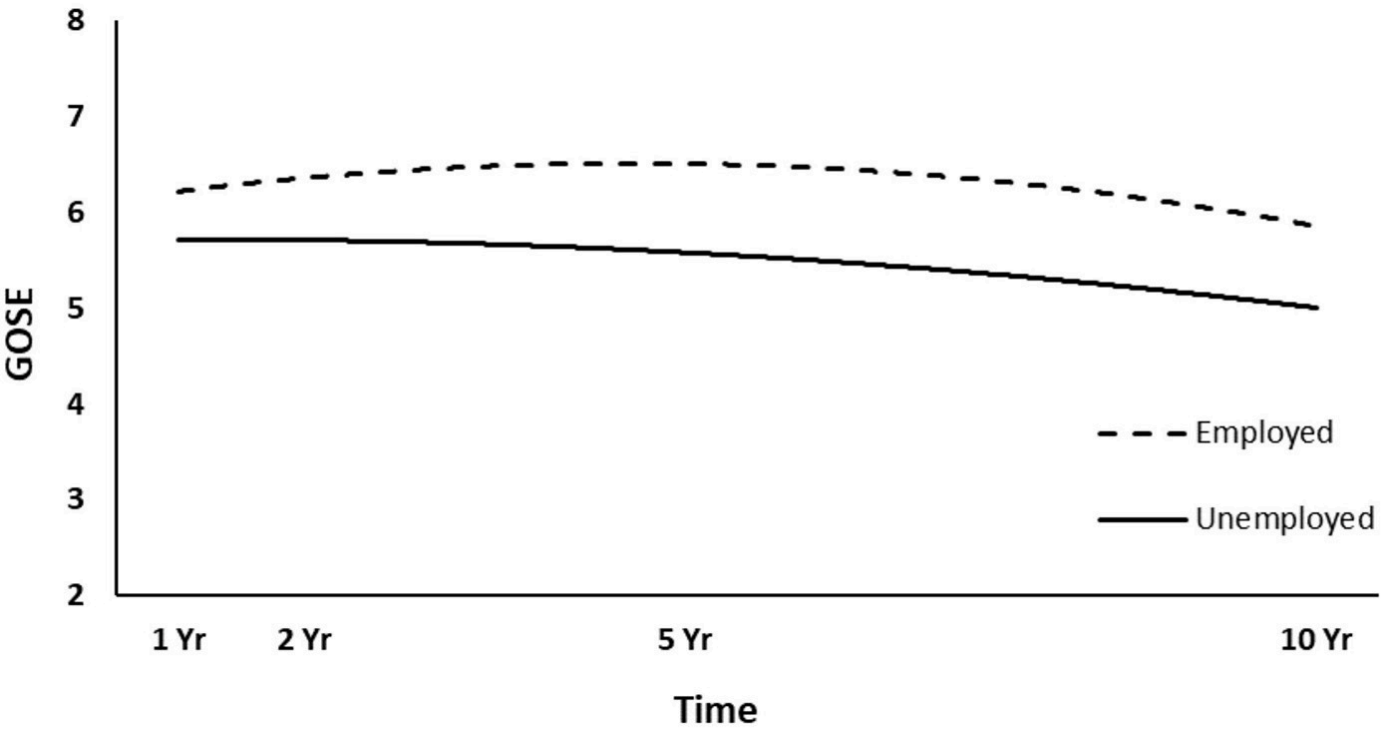
Main effect of employment at time of injury on GOSE trajectories.

**Figure 5:**
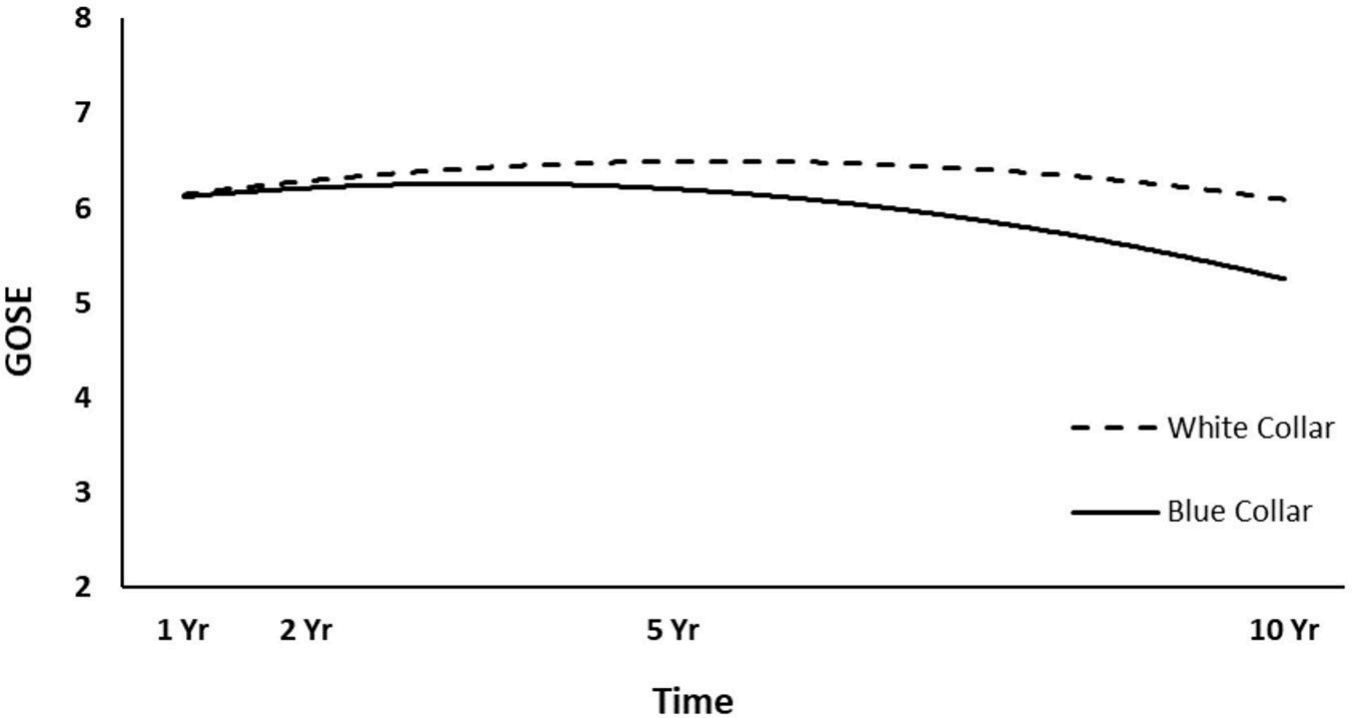
Main effect and quadratic time interaction effect of occupation type on GOSE trajectories.

**Figure 6:**
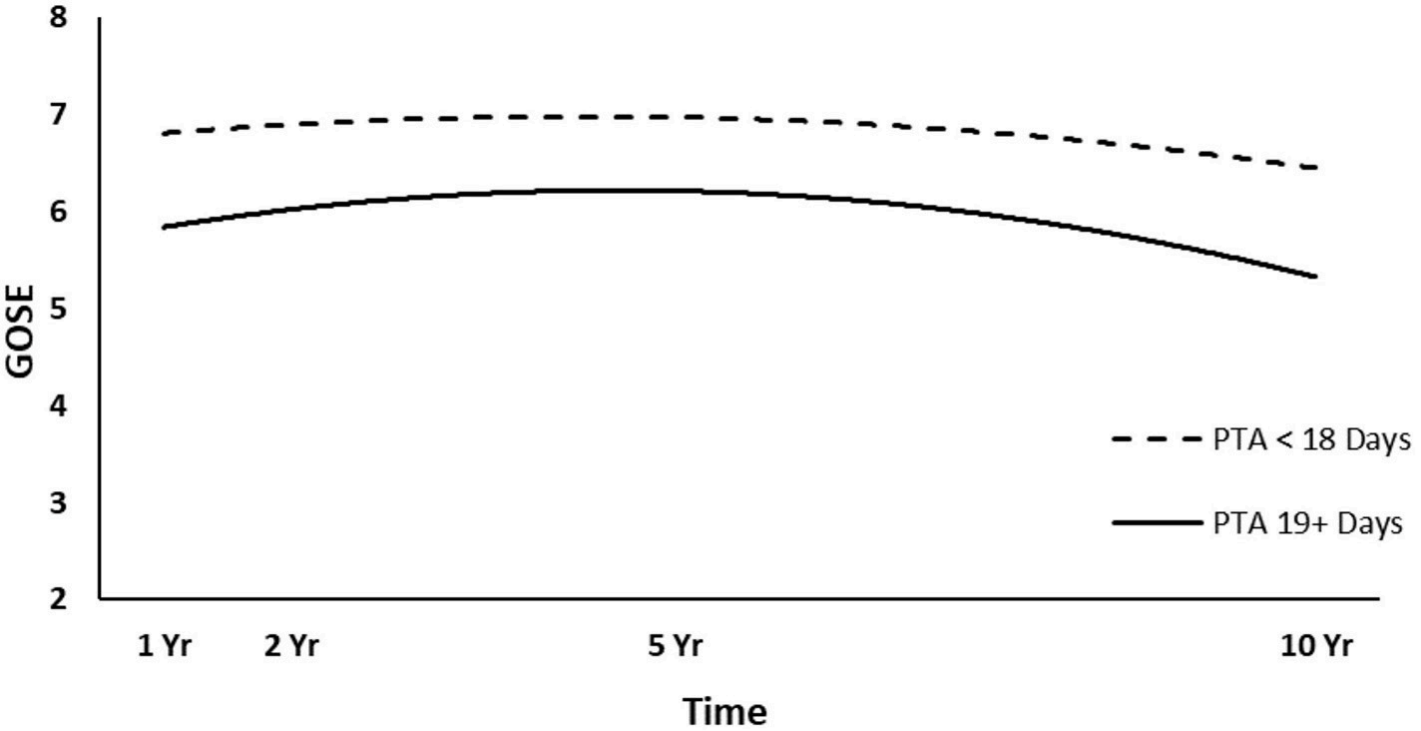
Main effect and quadratic time interaction effect of post-traumatic amnesia (PTA) duration (dichotomized at median value) on GOSE trajectories.

The full HLM examined whether socio-demographic and injury characteristics at baseline could predict the quadratic trajectories of GOSE scores over time. Table 4 shows all statistically significant and non-significant fixed effects from the full HLM and their b-weights, *p*-values, and 95% confidence intervals. The GOSE scores showed a significant quadratic trend over time, conforming to an initial increase and then decrease. Sex, age, employment at time of injury, occupation type, and length of PTA yielded statistically significant effects on the participants' GOSE trajectories. Men had higher GOSE quadratic trajectories across the four time points than women (Figure 2) (*p* = 0.013). Younger participants had higher GOSE quadratic trajectories than older participants (Figure 3) (*p* = 0.012). Participants who had been employed at time of injury had higher GOSE quadratic trajectories than those who had been unemployed (Figure 4) (*p* = 0.012). Participants in a white collar profession had higher GOSE quadratic trajectories than those in a blue collar profession (Figure 5) (*p* = 0.014). Finally, participants with a shorter PTA length had higher GOSE quadratic trajectories than those with a longer PTA duration (Figure 6) (*p* = 0.001).

**Table 4:** Socio-demographic and injury predictors of GOSE trajectories across 1, 2, 5, and 10 years after injury.

**Predictor**	**b-weight**	***SE***	***p*-value**	**95% confidence interval**	
				**Lower bound**	**Upper bound**
Intercept	5.90^***^	0.23	<0.0001	5.45	6.36
Time	0.016^**^	0.06	0.007	0.04	0.27
Sex (1 = woman, 0 = man)	−0.46^*^	0.18	0.013	−0.82	−0.10
Age	−0.02^*^	0.01	0.012	−0.04	−0.01
Relationship status (1 = partnered, 0 = single)	0.14	0.20	0.475	−0.25	0.53
Education	0.05	0.10	0.619	−0.15	0.25
Employment (1 = employed, 0 = unemployed)	0.51^*^	0.20	0.012	0.11	0.90
Occupation type (1 = white collar, 0 = blue collar)	0.43^*^	0.17	0.014	0.09	0.78
Glasgow coma scale score	0.02	0.03	0.383	−0.03	0.08
Cause of injury (1 = motor vehicle, 0 = not motor vehicle)	−0.29	0.17	0.099	−0.63	0.06
Post-traumatic amnesia	−0.01^**^	0.00	0.001	−0.02	0.00
CT severity score	−0.13	0.07	0.084	−0.28	0.02
Injury severity score	−0.01	0.01	0.405	−0.02	0.01
Time^*^time	−0.02^***^	0.01	<0.0001	−0.03	−0.01

*Full hierarchical model*.

* p < 0.05;

** p < 0.01;

*** *p < 0.0001*.

### Final HLM With Quadratic Time Interactions

The final HLM examined whether the previously significant predictors, as well as their interactions with quadratic time, could predict the quadratic trajectories of the GOSE scores. Table 5 shows all statistically significant and non-significant fixed effects from the final HLM and their b-weights, *p*-values, and 95% confidence intervals, although only the significant interaction terms will be focused on for interpretation. The significant time^*^time^*^occupation type interaction effect suggested that participants in a white collar profession tended to have a slightly increasing trajectory over the first 5 years, which curved back toward 1-year levels at 10 years (Figure 5). However, participants in a blue collar profession had a smaller increase in GOSE scores during the first 5 years, but a dramatic decrease in GOSE scores at 10 years, ending nearly a full point below their scores at the 1-year follow-up. The significant time^*^time^*^PTA interaction suggested that participants with shorter PTA duration had a slightly increased and then decreased trajectory over the 10 years, with GOSE scores at the final follow-up being somewhat lower than the scores at the 1-year follow-up (Figure 6). However, participants with longer PTA duration tended to have a sharper increase but then a more dramatic decrease in GOSE scores over the 10 years, ending with GOSE scores nearly half a point below their 1-year scores.

**Table 5:** Previously significant predictors and quadratic time interactions on GOSE trajectories across 1, 2, 5, and 10 years after injury.

**Predictor**	**b-weight**	***SE***	***p*-value**	**95% confidence interval**	
				**Lower bound**	**Upper bound**
Intercept	5.86	0.22	<0.0001	5.43	6.29
Time	0.17^**^	0.06	0.003	0.06	0.28
Sex (1 = woman, 0 = man)	−0.34	0.20	0.086	−0.73	0.05
Age	−0.01	0.01	0.095	−0.03	0.00
Employment (1 = employed, 0 = unemployed)	0.52^*^	0.22	0.018	0.09	0.95
Occupation type (1 = white collar, 0 = blue collar)	0.19	0.17	0.277	−0.15	0.53
Post-traumatic amnesia	−0.02^***^	0.00	<0.0001	−0.02	−0.01
Time^*^time	−0.03^***^	0.01	<0.0001	−0.04	−0.01
Time^*^time^*^sex	−0.01	0.00	0.194	−0.01	0.00
Time^*^time^*^age	0.00	0.00	0.597	0.00	0.00
Time^*^time^*^employment	0.00	0.00	0.982	−0.01	0.01
Time^*^time^*^occupation type	0.01^**^	0.00	0.001	0.00	0.02
Time^*^time^*^post-traumatic amnesia	0.00^*^	0.00	0.023	0.00	0.00

*Final hierarchical model*.

* p < 0.05;

** p < 0.01;

*** *p < 0.0001*.

## Discussion

This study is one of a few prospective studies to investigate the changes and predictors of global functioning in survivors of moderate to severe TBI over the first 10 years after injury. First, the distribution of GOSE categories over time showed dynamic changes, with improvement and deterioration over time. From the 5 to 10-year follow-up, approximately 7% of survivors improved one category, 56% showed no change, while 37% worsened one or two categories. Second, trajectory analysis using HLM suggested different global outcome trajectories within the cohort of survivors. Third, predictor analysis determined that sex, age, employment at time of injury, occupation type, and length of PTA yielded statistically significant effects on participants' GOSE trajectories. The findings provide insight in which TBI survivors face an increased risk of deterioration of global functioning over time, with the possibility of initiating tailored rehabilitation programs to attempt to counteract this development and to meet the long-term changing needs of this population.

When assessing changes in GOSE score categories between three consecutive follow-ups in the present study, there was a clear trend for more negative change toward the 10-year follow-up. Corrigan and Hammond (4) studied changes in GOSE score categories over four consecutive follow-ups (1–2, 2–5, 5–10, and 10–15 years after TBI). When looking at the development in GOSE scores in the 5–10-year follow-ups (*n* = 796), 42% of participants showed no change in GOSE score, whereas 24% improved one or two categories, and 34% deteriorated one or two categories. Compared to the present study, Corrigan and Hammond found a smaller proportion of participants with no change and a higher proportion of participants with improvement. However, similar to our results, a larger proportion of the survivors tended to experience deterioration in GOSE scores over time, supporting the concept of TBI as a chronic health condition (3).

McMillan et al. (39) followed survivors at 1, 5–7, and 12–14 years after mild to severe TBI (*n* = 87), where the GOSE score from 1 year to 12–14 years improved in 34% of survivors, remained the same in 32%, and worsened in 34%. These results are in line with our results from the 1 to 10-year follow-ups. However, they found that 23% of participants improved between the 5–7- and 12–14-year follow-ups, which is a much higher proportion compared to our study (7% from 5 to 10-year follow-up). Methodological differences between the two studies probably contributed to this discrepancy, where the study by McMillan have a high risk of selection bias due to significant drop out over time (*n* = 475 survivors assessed at 1-year follow-up, *n* = 87 survivors assessed at 12–14 years follow-up). In addition, a higher proportion of survivors with positive change can be expected in a study sample that included mild TBI. Andersson et al. (16) followed 61 survivors after severe TBI at 1 year and 10–15 years after injury with a stable GOS score between the time points, but reported that, in total, 15% of survivors had improved GOS scores, 55% showed no change, and 30% deteriorated. The more homogenous study sample of severe TBI (i.e., all requiring intracranial monitoring and artificial ventilation), as well as use of the GOS with fewer categories could perhaps explain a more stable functional outcome and less improvement over time as compared to our results.

The participants in the upper moderate disability and lower good recovery groups (GOSE score 6 and 7) had the largest negative change in GOSE scores from the 1- to 10-year follow-up. Our previous study on self-reported healthcare needs in survivors of moderate to severe TBI (49) found that survivors with GOSE scores of 6–8 (i.e., less severe disability) reported more unmet needs than survivors with GOSE scores of 2–5 (i.e., more severe disability) (38 vs. 13%). It was discussed that those with fewer problems may be more troubled by their problems and therefore report higher unmet needs, or perhaps this group is less prioritized for receiving healthcare services due to the assessed better outcome. We can only speculate whether the lack of healthcare services contributes to deterioration over time in this group.

Based on our previous studies (15, 17), we hypothesized that TBI-related global outcome would remain stable over the first 10 years after injury. Contrary to our hypothesis, the HLM of the quadratic GOSE score trajectories showed a significant change over time, with an initial increase and then decrease in GOSE scores up to 10 years after injury. These findings are partly consistent with two larger US studies looking at GOSE trajectories up to 20 years after TBI (18, 19), which found initial improvement in functional status before a peak, and a decline in GOSE scores. However, the decline started after the 10-year follow-up. It is possible that the socio-demographic and injury-related differences between study populations can explain these results; nonetheless, we could not make a closer comparison due to the limited reporting of such data in the US studies.

We found that TBI survivors who were male, younger, employed at time of injury, in a white collar occupation and with a shorter PTA duration (i.e., lower injury severity), had significantly higher global functioning across 1, 2, 5, and 10 years after moderate to severe TBI. Thus, the results are in agreement with our hypothesis.

Contrary to previous long-term studies (18, 19) and previous results reported from the present study sample (15), we found in the present study that men experience better functional trajectories up to 10 years after TBI. This is in line with a meta-analysis that found poorer outcomes in women for 85% of the measured outcome variables, including disability, after mild to severe TBI (27). Another review found inconclusive evidence of the gender effect on disability outcome, but most studies reported worse outcomes for women (20). Taken together, gender differences remain understudied and poorly understood in relation to TBI outcomes (55). The finding of better GOSE probability trajectories for younger survivors is consistent with a broad literature, which reports significantly better global functioning after TBI in younger survivors (15, 16, 18–21, 26). Return to work at different levels is captured through the representation of GOSE categories 5–8, from being able to work only with large adjustments/sheltered work to full functional recovery. A recent study of the present cohort has shown stable employment trajectories over 1, 2, 5, and 10 years after injury, with approximately half of the survivors returning to work (43). Numerous studies have shown that employment prior to injury is a strong predictor of return to work after moderate to severe TBI (46, 56, 57), which implies achieving a favorable outcome with regards to global function. In line with the present results, several studies have demonstrated a significant association between pre-injury employment and disability after TBI (15, 20, 31, 58). White collar occupation (i.e., professional, managerial, or administrative work) at the time of injury was a significant predictor of better GOSE trajectories up to 10 years after TBI. Interestingly, previous studies have not demonstrated this association, but it has been found to be a predictor of return to work (46, 59). In line with previous studies, lower injury severity (i.e., shorter PTA duration) was a significant predictor of better functioning trajectories at 1, 2, 5, and 10 years post-TBI (15, 29).

## Limitations and future directions

The present study has several limitations that should be acknowledged when interpreting the results. The inclusion criteria included survivors of moderate to severe TBI and aged 16–55 years; therefore, the results cannot be readily generalized to individuals with mild TBI or to individuals outside this age range. The participants were recruited through the Trauma Referral Centre and represent a mixed population with regards to the type and extent of inpatient rehabilitation received, and should therefore be representative of a broader range of patients than, for example, those in the TBIMS studies. Previous studies have hypothesized that trajectories of disability in elderly populations (aged >65 years) could differ from that of younger adult survivors of TBI (26), but we did not include that age group in this study.

The present study sample is small, and over time there has been an inevitable loss to follow-up. However, the attrition rate of 21% in the 1–10-year follow-up is low compared to that of other studies (13). The descriptive GOSE score changes should be interpreted with caution due to the missing data points and risk of selection bias. However, the HLM handles missing data well, and the longitudinal design with four follow-up time points (i.e., 388 observations) renders the trajectory analysis much stronger with regards to statistical power.

To sum up, further research is needed to verify the present study findings, preferentially through international collaboration to establish standardized research methodology and thereby generalizable knowledge on long-term functional outcome following TBI. This can for example be accomplished through multinational clinical TBI trials. Future studies should also incorporate a broader set of variables, such as physical, psychological, and cognitive functioning; personal traits; use of healthcare and rehabilitation services; as well as psychosocial support and lifestyle factors.

## Conclusions

This study aids understanding of the natural history of recovery following moderate to severe TBI by highlighting the trajectories of global functioning from the 1-year to 10-year follow-up, and examining predictors of better GOSE outcomes. The results suggests that more intensive and tailored rehabilitation programs may be required to counteract a negative global outcome development in survivors of older age, those unemployed at the time of injury and those with a longer PTA duration, as well as to address the long-term changing needs of this population.

## Data Availability

The dataset for this study will not be made publicly available. The dataset is not anonymous and cannot be shared publicly according to the Norwegian law on research ethics and medical research. The dataset can be reviewed on the grounds of Oslo University Hospital upon request, if considered appropriate and approved by the Regional Committees for Medical and Health Research Ethics (REC).

## Ethics Statement

The present study was conducted in accordance with the recommendations of the Norwegian law on research ethics and medical research. The protocol was approved by the Regional Committee for Medical and Health Research Ethics, East Norway, and the Norwegian Data Inspectorate. All subjects gave written informed consent in accordance with the Declaration of Helsinki.

## Author Contributions

MF, CR, SS, and NA contributed to the study design, data acquisition, analysis, interpretation, drafting, and finalizing of the manuscript. PP contributed to the analysis, interpretation, drafting, and finalizing of the manuscript. TH, SB, JL, and JA-L contributed to the data interpretation, drafting, and finalizing of the manuscript.

### Conflict of Interest Statement

The authors declare that the research was conducted in the absence of any commercial or financial relationships that could be construed as a potential conflict of interest.
